# Total thyroidectomy with ultrasonic dissector for cancer: multicentric experience

**DOI:** 10.1186/1477-7819-10-70

**Published:** 2012-04-27

**Authors:** Roberto Cirocchi, Carlo Boselli, Salvatore Guarino, Alessandro Sanguinetti, Stefano Trastulli, Jacopo Desiderio, Alberto Santoro, Fabio Rondelli, Giovanni Conzo, Domenico Parmeggiani, Giuseppe Noya, Giorgio De Toma, Nicola Avenia

**Affiliations:** 1 Department of General Surgery, S. Maria Hospital, University of Perugia, Terni, Italy; 2 Department of General and Oncologic Surgery, University of Perugia, Perugia, Italy; 3 University Department of Surgery, University College of London Hospitals (Royal Free), Pond Street, London, Greater London, NW3 2QG, United Kingdom; 4 Department of Surgical Sciences, Sapienza University of Rome, Rome, Italy; 5 General Surgery Unit, Second University of Naples, Naples, Italy; 6 Department of Gerontology, Geriatrics and Metabolic Disease, Second University of Naples, Naples, Italy; 7 Department of Surgery “P. Valdoni”, “Sapienza” University, Policlinico Umberto I, Rome, Italy

**Keywords:** Thyroidectomy, Ultrasonic dissector, Harmonic scalpel, Hypocalcaemia, Laryngeal nerve palsy, Thyroid cancer

## Abstract

**Background:**

We conducted an observational multicentric clinical study on a cohort of patients undergoing thyroidectomy for thyroid carcinoma. The aim of this study was to evaluate the benefits of the use of ultrasonic dissector (UAS) *vs.* the use of a conventional technique (vessel clamp and tie) in patients undergoing thyroid surgery for cancer.

**Methods:**

From June 2009 to May 2010 we evaluated 321 consecutive patients electively admitted to undergo total thyroidectomy for thyroid carcinoma. The first 201 patients (89 males, 112 females) presenting to our Department underwent thyroidectomy with the use of UAS while the following 120 patients (54 males, 66 females) underwent thyroidectomy performed with a conventional technique (CT): vessel clamp and tie.

**Results:**

The operative time (mean: 75 min in UAS *vs.* 113 min in CT, range: 54 to 120 min in UAS *vs.* 68 to 173 min in CT) was much shorter in the group of thyroidectomies performed with UAS. The incidence of transient laryngeal nerve palsy (UAS 3/201 patients (1.49%); CT 1/120 patients (0.83%)) was higher in the group of UAS; the incidence of permanent laryngeal nerve palsy was similar in the two groups (UAS 2/201 patients (0.99%) *vs.* CT 2/120 patients (1.66%)). The incidence of transient hypocalcaemia (UAS 17/201 patients (8.4%) *vs.* CT 9/120 patients (7.5%)) was higher in the UAS group; no relevant differences were reported in the incidence of permanent hypocalcaemia in the two groups (UAS 5/201 patients (2.48%) *vs.* 2/120 patients (1.66%)). Also the average postoperative length of stay was similar in two groups (2 days).

**Conclusion:**

The only significant advantage proved by this study is represented by the cost-effectiveness (reduction of the usage of operating room) for patients treated with UAS, secondary to the significant reduction of the operative time. The analysis failed to show any advantages in terms of postoperative transient complications in the group of patients treated with ultrasonic dissector: transient laryngeal nerve palsy (1.49% in UAS *vs.* 0.83% in CT) and transient hypocalcaemia (8.4% in UAS *vs.* 7.5%in CT). No significant differences in the incidence of permanent laryngeal nerve palsy (0.8% in UAS *vs.* 1.04% in CT) and permanent hypocalcaemia (2.6% in UAS *vs.* 2.04% in CT) were demonstrated. The level of surgeons’ expertise is a central factor, which can influence the complications rate; the use of UAS can only help surgical action but cannot replace the experience of the operator.

## Background

Theodor Billroth and Theodor Kocher pioneered the thyroid surgery at the end of the 19th century [1]. Throughout the 20th century innovations in this surgical specialty were quite modest. In 1999 P Miccoli introduced the minimally invasive video-assisted thyroidectomy (MIVAT) [2] and new instruments specifically designed for thyroidal vessels dissection were also invented (LigaSure and ultrasonic dissector). LigaSure is an energy generator which, by producing electric energy at high intensity and low voltage, coagulates vessels before sectioning them [3,4]. The ultrasonic dissector (UAS) cuts and coagulates at lower temperatures (50 to 100°C) compared with those reached by electric energy at high intensity or by laser (100 to 400°C). Also, the lateral thermic diffusion in the UAS is minor compared to the other coagulation instruments (approximately 2 mm). The first UAS launched in the market was the Harmonic ACE with 5 × 11 mm scissors [5]. A recent evolution of this was the Harmonic Focus which, with 9-mm scissors, was much more ergonomic and easier to handle than the first model [6]. Both instruments’ scissors are composed by a curved active blade facing another inert one with the only function to grasp the tissues. The quality of homeostasis depends on the amount of pressure exerted on the instrument and also on the level of ‘power output’ of the generator (level 3 for coagulation and level 5 for coagulation and cutting). It is extremely important not to use the active blade in the proximity of nervous structures and parathyroids for the high risk of damaging them. We have conducted an observational clinical study on a cohort of patients undergoing thyroidectomy for thyroid carcinoma. The main aim of this study was to evaluate the benefits of UAS *vs.* conventional technique (CT; vessel legation and tie) in patients undergoing thyroid surgery for primary thyroid malignancy.

## Methods

From June 2009 to May 2010 an observational study was conducted on 321 consecutive patients electively admitted to undergo thyroidectomy and central neck lymph node dissection for differentiated thyroid carcinoma (251 papillary thyroid carcinoma and 70 follicular thyroid carcinoma) (Table 1). Written informed consent was obtained from the patient for publication of this report and any accompanying images.

**Table 1:** Characteristics of patients

**Inclusion criteria**	**Exclusion criteria**	**Histopathologic features**
Patients with preoperative cytological or histological proved diagnosis of differentiated thyroid cancer	Patients with severe co-morbidities (severe obesity, diabetes, infective or hematological conditions) Patients on steroid or immunosuppressive medications Patients undergoing thyroidectomy and latero-cervical lymphadenectomy Patients undergoing secondary surgery in the cervical region Patients undergoing thyroidectomy for locally advanced tumors Patients with goitres submerged in the thorax	251 papillary thyroid carcinoma 70 follicular thyroid carcinoma

Inclusion criteria were: all patients with preoperative cytologically or histologically proved diagnosis of differentiated thyroid cancer.

Exclusion criteria were: patients with severe co-morbidities (severe obesity, diabetes, infective or hematological conditions); patients on steroid or immunosuppressive medications; patients undergoing thyroidectomy and latero-cervical lymphadenectomy; patients undergoing secondary surgery in the cervical region; patients undergoing thyroidectomy for locally advanced tumors; patients with goiters submerged in the thorax.

All patients were followed up in the outpatient clinic for a minimum of 1 year.

In this cohort study 321 patients were enrolled and allocated in the two groups only following a temporal criteria: the first 201 patients (89 males, 112 females) underwent thyroidectomy with UAS and the following 120 patients (54 males, 66 females) underwent thyroidectomy with a CT using traditional instruments by ligating and cut the vessels. The definitive histopathology examination confirmed the diagnosis of thyroid carcinoma in all of the patients (251 papillary and 70 follicular). Considering the TNM staging system we observed 199 patients with T1 tumors, 65 patients with T2 tumors, 43 patients with T3 tumors, and 14 patients with T4 tumors.

### Outcomes of interest

The following outcomes were used to compare the thyroidectomies performed using the UAS *vs.* the CT: operative time (minutes from the skin incision to skin closure); intraoperative blood loss (mL in the aspirator column); transient laryngeal nerve palsy (<6 months after surgery; number of patients); permanent laryngeal nerve palsy (>6 months after surgery; number of patients); transient hypocalcaemia (<6 months after surgery; number of patients); permanent hypocalcaemia (>6 months after surgery; number of patients); average postoperative length of stay; and wound infection rate.

On every patient, preoperative fibrolaryngoscopy was used to evaluate pre-existing conditions affecting the vocal cords functionality. Transient RILN palsy was assumed on patient with symptoms persisting for more than one week after surgery but resolving in less than six months. Permanent palsy was proved with laryngoscopy on patients with symptoms persisting for more than six months.

Wound infections were clinically detected on the basis of purulent discharge from the scar.

## Results and discussion

The studies in literature comparing thyroidectomy with UAS *vs.* CT are shown in Table 2.

**Table 2:** Studies in literature comparing thyroidectomy with UAS versus conventional technique

**Authors**	**Year**	**Total patients included (*n*)**	**UAS (*n*)**	**CT (*n*)**
Defechereux	2003	34	17	17
Ortega	2004	114	57	57
Cordòn	2005	66	29	37
Miccoli	2006	100	50	50
Hallgrimsson	2008	51	27	24
Lombardi	2008	200	100	100
Papavramidis	2009	90	45	45

Perioperative outcomes are summarized in Table 3. The operative time (mean: 75 min in UAS *vs.* 113 min in CT, range: 54 to 120 min in UAS *vs.* 68 to 173 min in CT) was considerably shorter in the group of thyroidectomies performed with UAS. The incidence of transient laryngeal nerve palsy (UAS 3/201 patients (1.49%) *vs.* CT 1/120 patients (0.83%)) was higher in the UAS group; the incidence of permanent laryngeal nerve palsy was similar in the two groups (UAS 2/201 patients (0.99%) *vs.* CT 2/120 patients (1.66%)). The incidence of transient hypocalcaemia (UAS 17/201 patients (8.4%) *vs.* CT 9/120 patients (7.5%)) was lower in the thyroidectomy conventionally performed; no statistically significant differences in the incidence of permanent hypocalcaemia (UAS 5/201 patients (2.48%) *vs.* 2/120 patients (1.66%)) were demonstrated between the two groups. There was a statistically significant reduction in the amount of blood loss (weighted mean difference (WMD), -60.10 mL; 95% confidence interval (CI), -117.04 to 3.16 mL) (*P* = 0.04) and of the overall volume of fluid collected in the surgical drains (WMD, -35.30 mL; 95% CI, -49.24 to 21.36 mL) (*P* = 0.00001) in the group of patients treated using the UAS. Data regarding the wound infection rate did not demonstrate any significant differences between the two groups. The average postoperative length of stay also was similar in two groups (2 days).

**Table 3:** Perioperative outcomes

**Type of thyroidectomy**	**Patients (*n*)**	**Operative time (mean)**	**Transient laryngeal nerve palsy**	**Permanent laryngeal nerve palsy**	**Transient hypocalcemia**	**Permanent hypocalcemia**	**Postoperative length of stay**
Ultrasonic dissector (UAS)	201	75 (54–120)	3	2	17	5	2
Conventional technique (CT)	120	113 (68–173)	1	2	9	2	2

Our study highlights that the main advantage brought by the use of UAS in thyroidectomies for malignant disease, is the shorter operating time (OT). This significant reduction in the OT was a result of the simultaneous coagulation and dissection exerted by the UAS; this technique resulted quicker compared to the repetitive ‘clip, cut and tie’ technique [7]. The same advantage was demonstrated also with the use of LigaSure. The reduction of OT allows also a significant reduction of costs by reducing the use of the operating room [8].

The incidence of both transient and permanent postoperative complications were higher in the UAS group (transient laryngeal nerve palsy: UAS 3/201 patients (1.49%) *vs.* CT 1/120 patients (0.83%); transient hypocalcaemia: UAS 17/201 patients (8.4%) *vs.* CT 9/120 patients (7.5%)) and it is similar in permanent complications (permanent laryngeal nerve palsy: UAS 2/201 patients (1%) *vs.* CT 2/120 patients (1.66%); permanent hypocalcaemia: UAS 5/201 patients (2.48%) *vs.* 2/120 patients (1.66%). Hypocalcaemia represents the most common postoperative complication after thyroidectomy [9]. In a study by Vaiman *et al.*, permanent recurrent laryngeal nerve palsy was observed in 1.4% and permanent hypocalcaemia in 3.5% in the TT group [10]. Re-intervention for recurrent goiter or for central neck dissection in thyroid cancer increases the risk of parathyroidectomy (*P* = 0.001); but there is no statistically significant difference in the incidence of postoperative hypocalcaemia (*P* = 0.55) [11]. A systematic review of the adverse effects of thyroidectomy combined with central neck dissection compared with thyroidectomy alone did not demonstrate increased risk of hypoparathyroidism by performing the two procedures at the same time [12]. Another systematic review was undertaken for the diagnosis of recurrent laryngeal nerve palsy after thyroidectomy: the average incidence of temporary IRLN palsy after thyroid operations was 9.8% and the incidence of permanent IRLN palsy was 2.3%. In their systematic review, Jeannon *et al.* encountered a variable IRLN palsy rate according to the method of examining the larynx and ranging from 2.3% to 26% [13]. While new techniques are continuously proposed to avoid nervous lesions of larynx and parathyroid (intraoperative laryngeal nerve monitoring during thyroidectomy, routine intraoperative rapid parathyroid hormone monitoring) [14-17] the experience of the surgeon and the choice of surgical technique represent important aspects to avoid hypocalcaemia and laryngeal nerve palsy. Although increasing interest is growing on the development of new techniques to prevent nervous lesions and parathyroid injuries (intraoperative RILN monitoring, intraoperative rapid parathyroid hormone monitoring) [14-17], we believe surgeons’ skills and technical choices represent the most important factor to prevent hypocalcaemia and IRLN injuries.

Kocher had already highlighted this problem: ‘Since we have adhered strictly to this procedure, the hoarseness, formerly so frequently observed after operation, has now become exceptional’. Subsequently in 1919 Halsted wrote in ‘The Operative Story of Goitre’: ‘Kocher, neat and precise, operating in a relatively bloodless manner, scrupulously removed the entire gland, doing little damage outside its capsule, Billroth, operating more rapidly, and, as I recall his manner, with less regard for the tissues and less concern for the hemorrhage, might easily have removed the parathyroids or at least have interfered with their blood supply, and have left fragments of the thyroid’ [18].

In the systematic review of Rouwen *et al*., on the best surgical approach for treating multinodular goiter [19], the use of an UAS does not seem to influence complication rates as reported in two studies [20,21]; these conclusions are in line with our meta-analysis on ‘thyroidectomy with ultrasonic dissector versus conventional clamp and tie’ [22]. Defechereux *et al.* in their prospective randomized study demonstrated that the essential advantages of the ultrasonic dissector were a significant shorter OT, decreased amount of blood loss and reduction of parathyroid injury [23]. This last result is not entirely in line with our study.

Our study shows a relevant advantage of cost-effectiveness in patients treated with UAS; there is a statistically significant reduction in the OT (WMD, -18.74 min; 95% CI, -26.97 to −10.52 min) (*P* = 0.00001), intraoperative blood loss (WMD, -60.10 mL; 95% CI, -117.04 to 3.16 mL) (*P* = 0.04) and overall drainage volume (WMD, -35.30 mL; 95% CI, -49.24 to 21.36 mL) (*P* = 0.00001) in the patients underwent thyroidectomy with UAS. Our analysis showed that the patients who were treated with UAS did not present more favorable results in incidence of postoperative transient complications but similar results about permanent complications (transient laryngeal nerve palsy: *P* = 0.11; permanent laryngeal nerve palsy: not estimable; transient hypocalcaemia: *P* = 0.24; permanent hypocalcaemia: *P* = 0.45).

## Conclusions

The only significant advantage shown in the present study is represented by an improved cost-effectiveness secondary to a reduction of the usage of operating room space for patients treated with UAS, due to a significant reduction of operative time. Our analysis showed that the patients who were treated with UAS did not present more favorable results in terms of postoperative transient complications: transient laryngeal nerve palsy (1.49% in UAS *vs.* 0.83% in CT) and transient hypocalcaemia (8.4% in UAS *vs.* 7.5% in CT). There was no significant difference in the incidence of permanent laryngeal nerve palsy (0.8% in UAS *vs.* 1.04% in CT) and permanent hypocalcaemia (2.48% in UAS *vs.* 1.66% in CT). The level of surgeons’ experience is an important factor which can influence the onset of these complications; the use of UAS can only help surgical action but cannot replace the experience of the surgeon.

## Abbreviations

CI = Confidence interval; CT = Conventional technique; OT = Operating time; UAS = Ultrasonic dissector; WMD = Weighted means difference.

## Competing interests

The authors declare that they have no competing interests.

## Authors’ contributions

All authors read and approved the final manuscript.
